# Sex chromosome and gonadal hormone contributions to binge-like and aversion-resistant ethanol drinking behaviors in Four Core Genotypes mice

**DOI:** 10.3389/fpsyt.2023.1098387

**Published:** 2023-03-07

**Authors:** Elizabeth A. Sneddon, Brianna M. Masters, Kiara D. Ream, Kaila A. Fennell, Jenelle N. DeMedio, Miranda M. Cash, Brynn P. Hollingsworth, Sai Pandrangi, Chloe M. Thach, Haifei Shi, Anna K. Radke

**Affiliations:** ^1^Department of Psychology, Miami University, Oxford, OH, United States; ^2^Center for Neuroscience and Behavior, Miami University, Oxford, OH, United States; ^3^Department of Biology, Miami University, Oxford, OH, United States

**Keywords:** aversion-resistant drinking, binge alcohol drinking, reward seeking, mouse model, operant responding, drinking in the dark, four core genotypes

## Abstract

**Introduction:**

While substantial research has focused on the contribution of sex hormones to driving elevated levels of alcohol drinking in female rodents, fewer studies have investigated how genetic influences may underlie sex differences in this behavior.

**Methods:**

We used the Four Core Genotypes (FCG) mouse model to explore the contribution of sex chromosome complement (XX/XY) and gonad type [ovaries (*Sry–*)/testes (*Sry+*)] to ethanol (EtOH) consumption and quinine-resistant drinking across two voluntary self-administration tasks: limited access consumption in the home cage and an operant response task.

**Results:**

For limited access drinking in the dark, XY/*Sry +* (vs. XX/*Sry* +) mice consumed more 15% EtOH across sessions while preference for 15% EtOH vs. water was higher in XY vs. XX mice regardless of gonad type. XY chromosomes promoted quinine-resistant drinking in mice with ovaries (*Sry–*) and the estrous cycle did not affect the results. In the operant response task, responding for EtOH was concentration dependent in all genotypes except XX/*Sry* + mice, which maintained consistent response levels across all concentrations (5–20%) of EtOH. When increasing concentrations of quinine (100–500 μM) were added to the solution, FCG mice were insensitive to quinine-punished EtOH responding, regardless of sex chromosome complement. *Sry* + mice were further found to be insensitive to quinine when presented in water. Importantly, these effects were not influenced by sensitivity to EtOH’s sedative effect, as no differences were observed in the time to lose the righting reflex or the time to regain the righting reflex between genotypes. Additionally, no differences in EtOH concentration in the blood were observed between any of the genotypes once the righting reflex was regained.

**Discussion:**

These results provide evidence that sex chromosome complement regulates EtOH consumption, preference, and aversion resistance and add to a growing body of literature suggesting that chromosomal sex may be an important contributor to alcohol drinking behaviors. Examination of sex-specific genetic differences may uncover promising new therapeutic targets for high-risk drinking.

## 1. Introduction

Female subjects have historically been excluded from clinical and preclinical research studies (1). To address this inequity and the lack of knowledge on women’s health, the scientific community has been pushing for inclusion of female subjects. In the United States about 15 million people have alcohol use disorder (AUD), including about 9.2 million men and 5.3 million women (2), and high-risk drinking behavior in women is on the rise (3). In the past 16 years, the prevalence of AUD has narrowed between men and women (4) and studies in both humans (3–6) and rodents (7–9) suggest that females may be more vulnerable to alcohol than males. However, there is still relatively little known regarding sex differences in the neurobiological mechanisms that drive drinking behaviors.

When considering sex differences in behavior, gonadal hormones and sex chromosomes are the two main contributors. In regard to hormones, estrogens are known to facilitate alcohol drinking (10–16) while progesterone may be protective against the development of alcohol-related behaviors in females (17). Investigations of the influence of chromosomal sex on sex differences in addictive behavior are lacking, primarily due to the difficulty of separating chromosomal sex from gonadal sex. Because the sex determining gene (SRY) is located on the Y chromosome, chromosomal sex (XX vs. XY) typically determines gonadal sex (ovaries vs. testes), and investigation of the independent influence of sex chromosomes on behavior is not possible.

Despite methodological difficulties, there are previous studies suggesting that sex chromosome complement influences alcohol drinking behaviors. These studies used the Four Core Genotypes (FCG) mouse model, which, due to translocation of SRY to an autosome, allows the influence of chromosomal and gonadal sex on behavior to be assessed independently (18). The first demonstration of a sex chromosome complement effect found greater habitual responding for ethanol (EtOH) in XY vs. XX mice, regardless of gonadal status (19). We have further demonstrated heightened EtOH consumption and preference in mice with the XX vs. XY chromosome complement using a continuous access drinking paradigm (20). When assessing relapse susceptibility, we found that only mice with the XX chromosome complement increased EtOH consumption following a series of deprivations (20). A recent preprint shows that chromosome complement influences binge-like EtOH drinking by interacting with gonadal status in a three-bottle drinking in the dark (DID) paradigm (21). These data suggest that genes on the X and Y chromosomes may be important regulators of alcohol-related behaviors.

The current studies were designed to investigate the role of sex chromosomes in alcohol-related behaviors, particularly aversion-resistance, and further characterize alcohol-drinking behaviors in the FCG line. FCG mice were tested using two commonly employed behavioral approaches: a two-bottle choice limited access paradigm frequently referred to as DID and an operant response paradigm. In both tasks, the bitter tastant quinine was used to model aversion-resistant drinking, which has been shown to be greater in female vs. male rodents under some (22–25), but not all experimental conditions (26, 27). In the limited access study, the estrous cycle was monitored in *Sry–* (ovaries) mice. Sensitivity to quinine and EtOH’s sedative effects were also assessed in separate cohorts of control mice. Together, the results of these studies suggest that chromosomal sex influences to the development of binge-like and aversion-resistant alcohol drinking behaviors in mice.

## 2. Materials and methods

### 2.1. Subjects

Two hundred and forty-nine FCG mice (PND 60+) were bred from breeding pairs consisting of *Sry +* male and C57BL/6 J (wild type) female mice at the Laboratory of Animal Resources at Miami University. Four groups were bred: XX/*Sry–*, XY/*Sry–*, XX/*Sry+*, and XY/*Sry+*. For genotyping, mice were lightly anesthetized with isoflurane and then ear tissue samples were collected and sent to Transnetyx (Cordova, TN).

In adulthood, mice were transferred to a temperature-controlled room. For the home cage drinking experiment, they were kept in a room that was on a 12:12 h reverse light:dark cycle (i.e., lights off at 7 AM). For the operant experiments, mice were kept in a room that was on a 12:12 light:dark cycle (i.e., lights on at 7 AM). Prior to experimentation, mice were group housed by gonad type (ovaries or testes). For the home cage drinking experiment, at least 3 days before experimentation, mice were individually housed in standard shoe box udel polysufone rectangular mouse cage (18.4 × 29.2 × 12.7 cm) with 5.08 × 5.08 cm nestlets and Bed-O-Cobb 0.634 cm bedding (Cincinnati Lap Supply, Cincinnati, OH). All mice received standard care and had access to Rodent Diet 5,001 chow (Cincinnati Lap Supply, Cincinnati, OH, United States) and reverse-osmosis (RO) drinking water *ad libitum* unless specified otherwise. All mice were cared for in agreement with the guidelines set by the National Institutes of Health and all procedures were approved by the Institutional Animal Care Use Committee (IACUC) at Miami University.

### 2.2. Drinking solutions

EtOH was prepared volume/volume in RO water at concentrations of 5, 10, 15, and 20%. Sucrose was prepared weight/volume in RO water at concentrations of 5 and 10%. Quinine hemisulfate (Q1250-50G, Millipore-Sigma, ST. Louis, MO, United States) solutions were prepared as 100, 250, and 500 μM in EtOH or RO water. All solutions were made fresh prior to each testing session.

### 2.3. Limited access home-cage EtOH drinking in the dark

**Figure 1:**
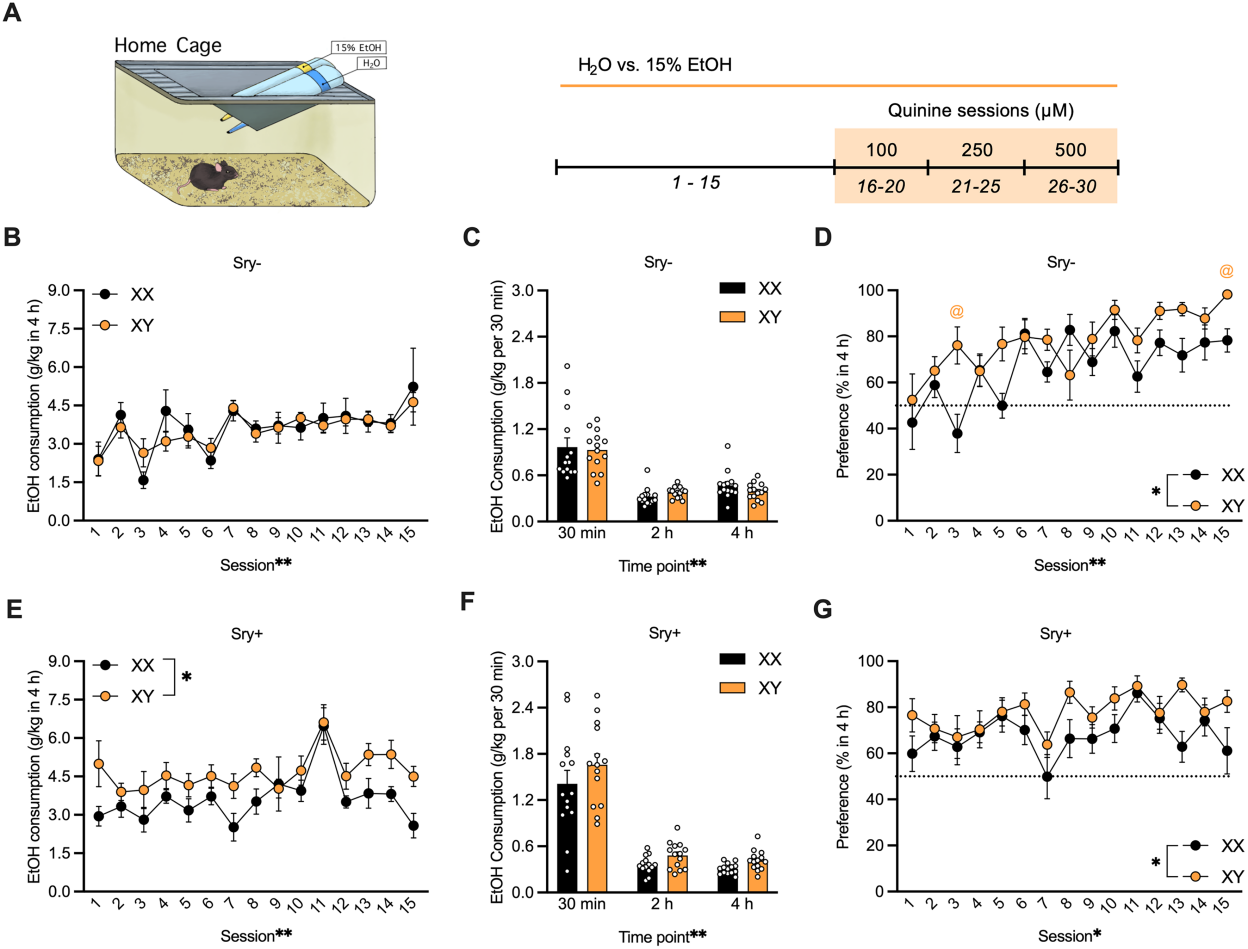
Home-cage consumption and preference in four core genotypes (FCG) mice. **(A)** Mice (XX/*Sry –*: *n* = 14, XY/*Sry –*: *n* = 14, XX/*Sry +*: *n* = 14, and XY/*Sry +*: *n* = 14) had access to 15% ethanol (EtOH) or water across 15 drinking sessions. Increasing concentrations of quinine (100, 250, and 500 μM) were added to the EtOH bottle for five sessions/concentration. The estrous cycle was monitored on the last four sessions of each quinine concentration in mice with ovaries (*Sry–*). Bottles were weighed at the 30 min, 2, and 4 h timepoints. Sex chromosomes do not influence 15% EtOH consumption **(B)** across session or **(C)** front-loading drinking behavior in *Sry–* mice. To assess front-loading behavior for EtOH, each timepoint was normalized to match consumption in 30 min using the following calculations: 2-h timepoint as ((*2-h timepoint–30-min timepoint*)/3) and 4-h timepoint as ((*4-h timepoint–2-h timepoint*)*/4*). **(D)** Preference for 15% EtOH vs. water is higher in *Sry –* mice with XY chromosomes across session. **(E)** Intake of 15% across session is higher in XY vs. XX *Sry* + mice but **(F)** front-loading drinking behavior is not altered. **(G)** Preference for 15% EtOH vs. water is higher in *Sry +* mice with XY chromosomes across session. All data expressed as mean ± standard error of the mean (SEM). ^*^p < 0.05, ^**^p < 0.01 (main effect, Two-Way RM ANOVA).

Mice (XX/*Sry–*: *n* = 14, XY/*Sry–: n* = 14, XX/*Sry+: n* = 14, and XY/*Sry+*: *n* = 14) were given access to water and 15% EtOH 3 h into the dark:light cycle (i.e., 10 AM) in a two-bottle, limited access DID paradigm for 4 h and for a total of 15 drinking sessions (Figure 1A). Bottles were weighed at 30 min, 2, and 4 h. Aversion-resistant drinking was tested by adding increasing quinine concentrations from 100, 250 to 500 μM for 5 drinking sessions per concentration (i.e., 15 quinine sessions total). Mice were weighed prior to testing and bottles were alternated daily to equate side biases. Mice with ovaries (*Sry–*) were scruffed two times a week following drinking to habituate them to the scruffing process (28, 29). *Sry–* mice and *Sry +* mice were tested in separate cohorts.

### 2.4. Estrous cycle monitoring

**Figure 2:**
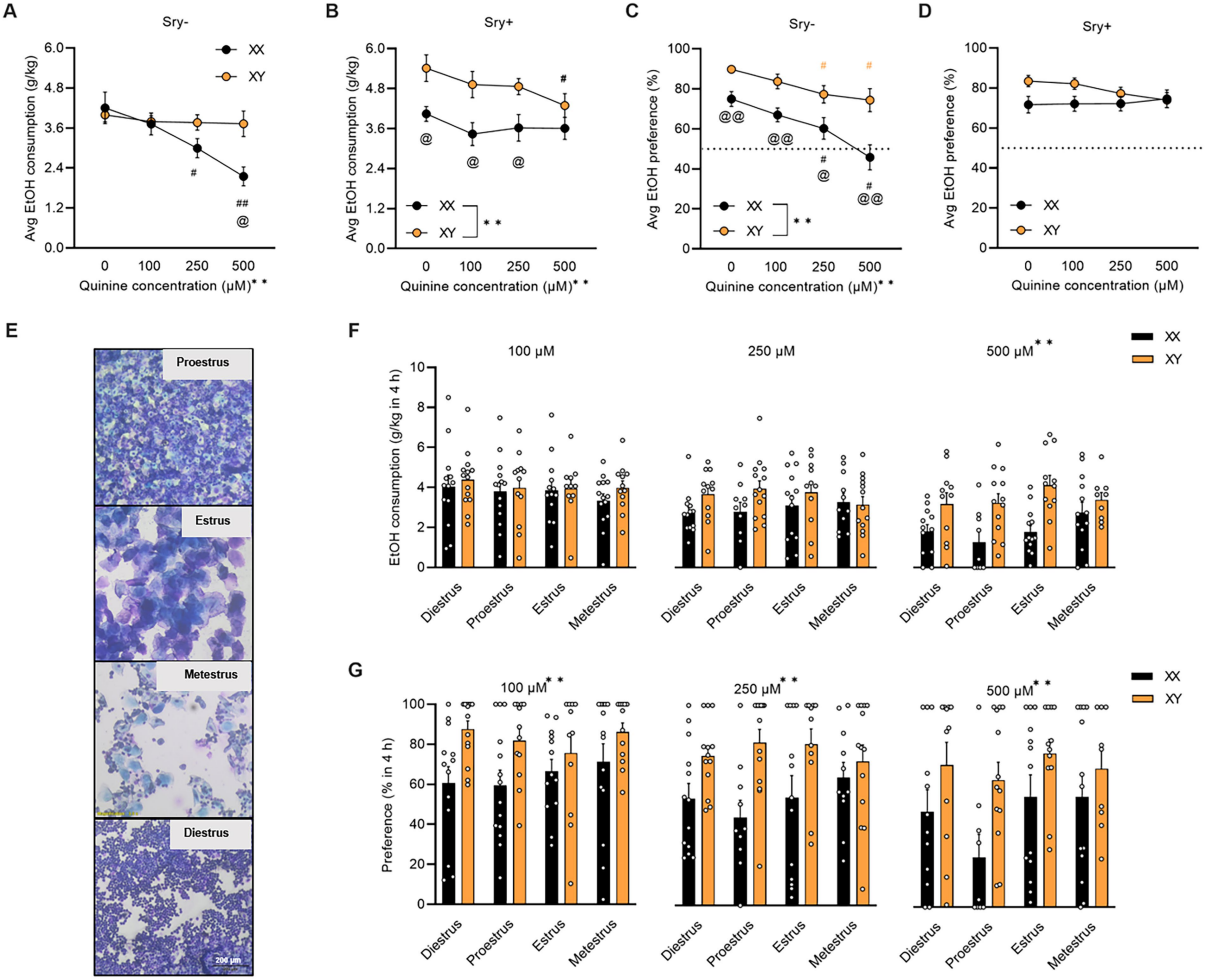
Aversion-resistant home-cage drinking in FCG mice. **(A)** Average aversion-resistant consumption is greater in *Sry–* mice with XY chromosomes (XX/*Sry–*: *n* = 14, XY/*Sry–*: *n* = 14) and **(B)** in *Sry +* mice with XX chromosomes (XX/*Sry +: n* = 14, and XY/*Sry +*: *n* = 14). **(C)** Average preference for quinine + 15% EtOH is greater in *Sry–* mice with XY chromosomes. **(D)** Sex chromosomes do not influence average preference for quinine + 15% EtOH in *Sry* + mice. **(E)** Visual representation of vaginal lavage cytology at 3.5 × magnification from *Sry–* mice in proestrus, estrus, metestrus, and diestrus. **(F)** Average EtOH intake across session at 100, 250, and 500 μM quinine + EtOH did not differ between phases of the estrous cycle. Individual data points show average consumption for each individual subject. **(G)** Average preference across session for 100, 250, and 500 μM quinine + EtOH did not vary by estrous phase but mice with the XY chromosomes complement show greater preference across all concentrations. Individual data points show average preference for each individual subject. All data expressed as mean ± standard error of the mean (SEM). ^#^*p* < 0.05 vs. 0 μM, ^##^*p* < 0.01 vs. 0 μM (Dunnett’s), ^@^*p* < 0.05 XX vs. XY, ^@@^*p* < 0.01 XX vs. XY (Holm Sidak’s), ^**^p < 0.01 (main effect of chromosome complement, Two Way RM or Mixed Effects ANOVA).

The estrous cycle was monitored on the last 4 sessions for each quinine concentration in the home-cage drinking experiment. Vaginal cells were collected in mice with ovaries (*Sry–*) 10 min before bottles were placed on cages. The sample air-dried for 24 h and was then stained with a DipQuick staining kit (30-s/solution; Jorgensen Laboratories, Loveland, CO). Determination of the phase of the cycle was based on the pattern of cell types of vaginal smear samples (Figure 2E) using a Nikon Labophot Phase Contrast light microscope (Diagnostic Instruments, Inc., Sterling Heights, MI) (30).

### 2.5. Operant apparatus

The operant procedures used were similar to those used in Sneddon et al. (2020). The conditioning chambers measured 6″ × 5.25″ × 5″ (Med Associates, Fairfax, VT, ENV-307A). The grid floor was made of 19 metal rods separated by 0.31″ (Med Associates, ENV-307A-GFW). Reward and activity responses were recorded through 2 nose poke holes located on one wall of the chamber. The left hole was considered active and resulted in reward delivery, while the right was inactive. A reward receptacle located on the same wall between the nose poke holes dispensed grain pellets (Bio-Serv, Flemington, NJ, F05684) from a pedestal-mounted pellet dispenser (Med Associates, ENV-203-20) and liquid solutions from a 20-ml syringe. Following a nose poke on the active side, 50 μl of solution was pushed into a well inside the reward receptacle *via* a single speed syringe pump (Med Associates, PHM-100) over 1.5 s. The wells were checked at the end of every session to confirm consumption. A light fixed in the reward receptacle turned on at the start of each session and remained on for the entire 30 min session (Med Associates, ENV-303RL). The rest of the chamber remained dark.

### 2.6. Response training for EtOH

Food restriction, to increase stable engagement with the operant task (31), began 2 weeks prior to test and continued during operant testing to maintain body weights at 85% of each mouse’s free-feeding weight. Mice were given measured quantities of food once daily after sessions. Training and testing sessions took place once daily from Monday to Friday 3 h into the light cycle (i.e., 10 AM). All training sessions lasted for 30 min and testing chambers were cleaned with 70% EtOH in between each session. The well was checked after each session to ensure there was no liquid remaining.

The first 3 days of training consisted of the mice responding for a grain pellet on a fixed ratio (FR) 1 schedule. A sucrose fading procedure was implemented on session 4. This procedure facilitates responding and encourages the intake of EtOH over the course of operant conditioning by first introducing a high-value reward (32–36). Mice responded for 10% sucrose on an FR1 schedule for 3 sessions followed by FR3 for 3 sessions. For all following sessions, mice responded on an FR3 schedule. Then, 10% EtOH was added to the solution and sucrose was faded out. For the experiment assessing responding for escalating concentrations of EtOH, mice were presented with the following solutions during response training: 10% sucrose, 10% sucrose + 10% EtOH, and 5% sucrose + 5% EtOH. For the experiment assessing responding for quinine-adulterated EtOH, mice were presented with the following solutions during response training: 10% sucrose, 10% sucrose +10% EtOH, and 5% sucrose +10% EtOH. Each solution was administered over a minimum of 3, but no more than 5, sessions. If the responses produced a coefficient of variation <20% after 3 consecutive sessions, the mice were moved on to the next solution.

### 2.7. Responding for escalating concentrations of EtOH

**Figure 3:**
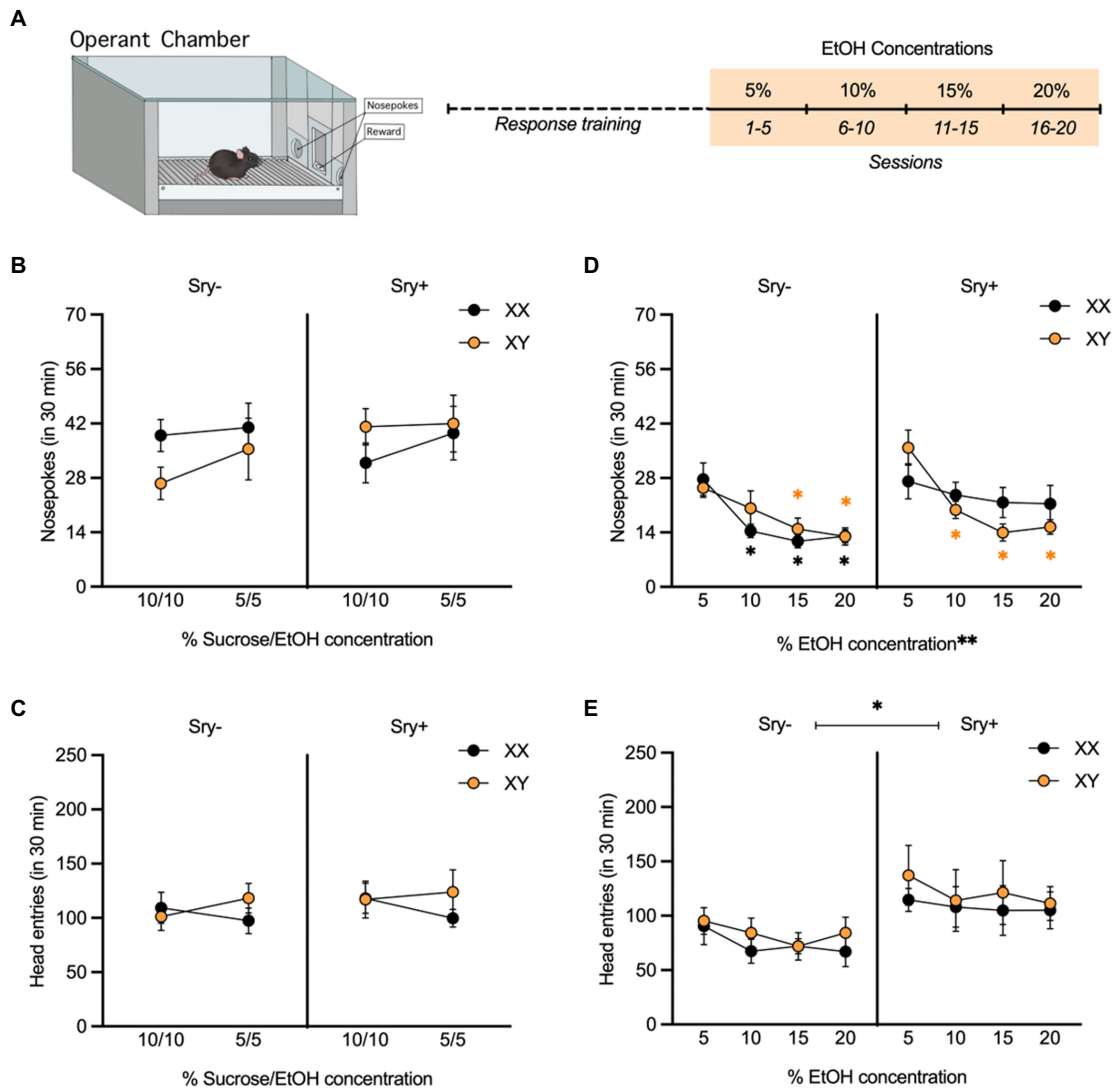
Operant responding for escalating EtOH concentrations in FCG mice. **(A)** Mice (XX/*Sry –*: *n* = 10, XY/*Sry –*: *n* = 10, XX/*Sry +: n* = 10, and XY/*Sry +*: *n* = 10) were trained to respond for EtOH on an FR3 schedule using a sucrose fading procedure followed by escalating concentrations of EtOH (5, 10, 15, and 20%) for 5 sessions each. **(B)** On average, mice responded for the 10% sucrose + 10% EtOH and 5% sucrose + 5% EtOH solutions during response training, but there were no differences between genotypes. **(C)** Head entries into the reward receptacle did not differ between the genotypes or with concentration. **(D)** The average number of nosepokes made for higher concentrations of EtOH decreased in all genotypes except XX/*Sry* + mice. ^*^p < 0.05 vs. 5% (Holm-Sidak’s), ^**^p < 0.01 (main effect, Three-Way ANOVA). **(E)** *Sry* + mice made more entries into the reward receptacle when responding for EtOH compared to *Sry* – mice. ^*^p < 0.05 (main effect, Three-Way ANOVA). All data expressed as mean ± standard error of the mean (SEM).

Responses for escalating concentrations of EtOH in the operant chamber were assessed in FCG mice (XX/*Sry–: n* = 10, XY/*Sry–: n* = 10, XX/*Sry+: n* = 10, and XY/*Sry+*: *n* = 10). Following response training, responses for escalating concentrations of EtOH were assessed as follows: 5% EtOH, 10% EtOH, 15% EtOH, and 20% EtOH (each for 5 sessions). Two mice (XX/Sry + = 1, XY/Sry– = 1) accidentally received 10% EtOH for five sessions before the 5% concentration was introduced (Figure 3A), but as their responses were similar to other mice of their genotype they were included in the analysis.

### 2.8. Quinine- or footshock-resistant EtOH responding

**Figure 4:**
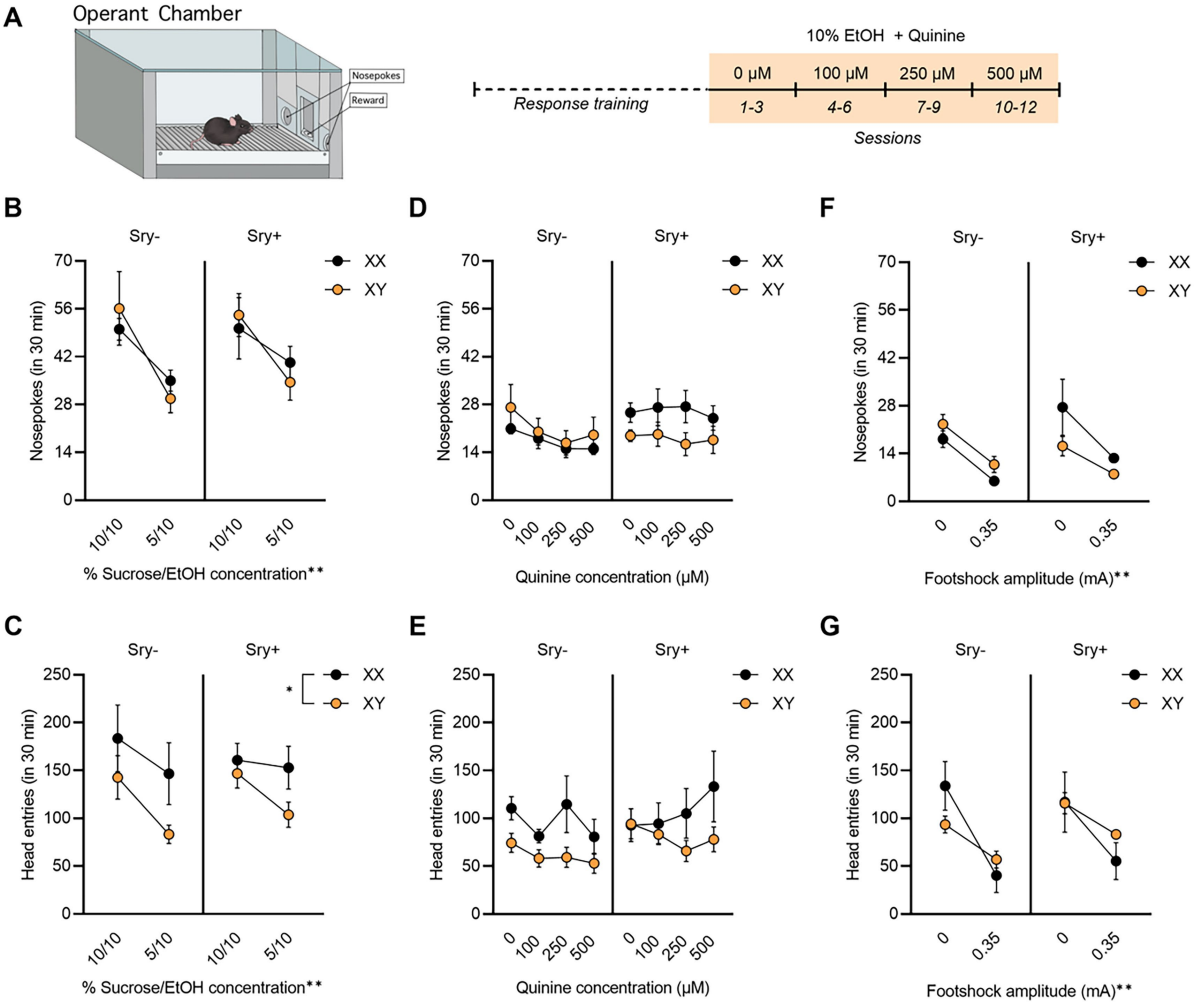
Operant responding for EtOH + quinine in FCG mice. **(A)** Mice (XX/*Sry –*: *n* = 10, XY/*Sry –*: *n* = 10, XX/*Sry +: n* = 11, and XY/*Sry +*: *n* = 9) were trained to respond for EtOH on an FR3 schedule using a sucrose fading procedure followed by 10% EtOH with escalating concentrations of quinine for three sessions each. **(B)** On average, mice responded less for the 5% sucrose + 10% EtOH solution during response training, but there were no differences between genotypes. **(C)** Head entries into the reward receptacle decreased for the 5% sucrose + 10% EtOH solution and XX mice made more head entries than XY mice. **(D)** FCG mice did not reduce average responses for 10% EtOH at any concentration of quinine. **(E)** Head entries into the reward receptacle did not decrease when quinine was added to the solution. **(F)** Nosepokes and **(G)** head entries decreased in all genotypes when 0.35 mA footshock was paired with responding for EtOH in a subset of mice (XX/*Sry–*: *n* = 3, XY/*Sry–*: *n* = 5, XX/*Sry +: n* = 3, and XY/*Sry +*: *n* = 2). ^*^p < 0.05, ^**^p < 0.01 (main effect, Three–Way ANOVA). All data expressed as mean ± standard error of the mean (SEM).

Responses for 10% EtOH adulterated with escalating concentrations of quinine were assessed in a separate cohort of FCG mice (XX/*Sry–*: *n* = 12, XY/*Sry–*: *n* = 19, XX/*Sry+: n* = 13, and XY/*Sry+*: *n* = 14). Following response training, mice responded for 10% EtOH for a minimum of 3 and a maximum of 5 sessions, depending on the previously mentioned coefficient of variation threshold. The mice were then given increasing solutions of 10% EtOH + quinine for 3 sessions each as follows: 10% EtOH +100 μM quinine, 10% EtOH +250 μM quinine, and 10% EtOH + 500 μM quinine (Figure 4A). Partway through the experiment, once it became apparent that FCG mice were potentially less sensitive to quinine, a final footshock session was added to the protocol. Thus, in a subset of mice (XX/*Sry–*: *n* = 3, XY/*Sry–*: *n* = 5, XX/*Sry+: n* = 3, and XY/*Sry+*: *n* = 2), responding for 10% EtOH was paired with a 0.35 mA footshock on one final test session.

### 2.9. Limited access home-cage water drinking in the dark

**Figure 5:**
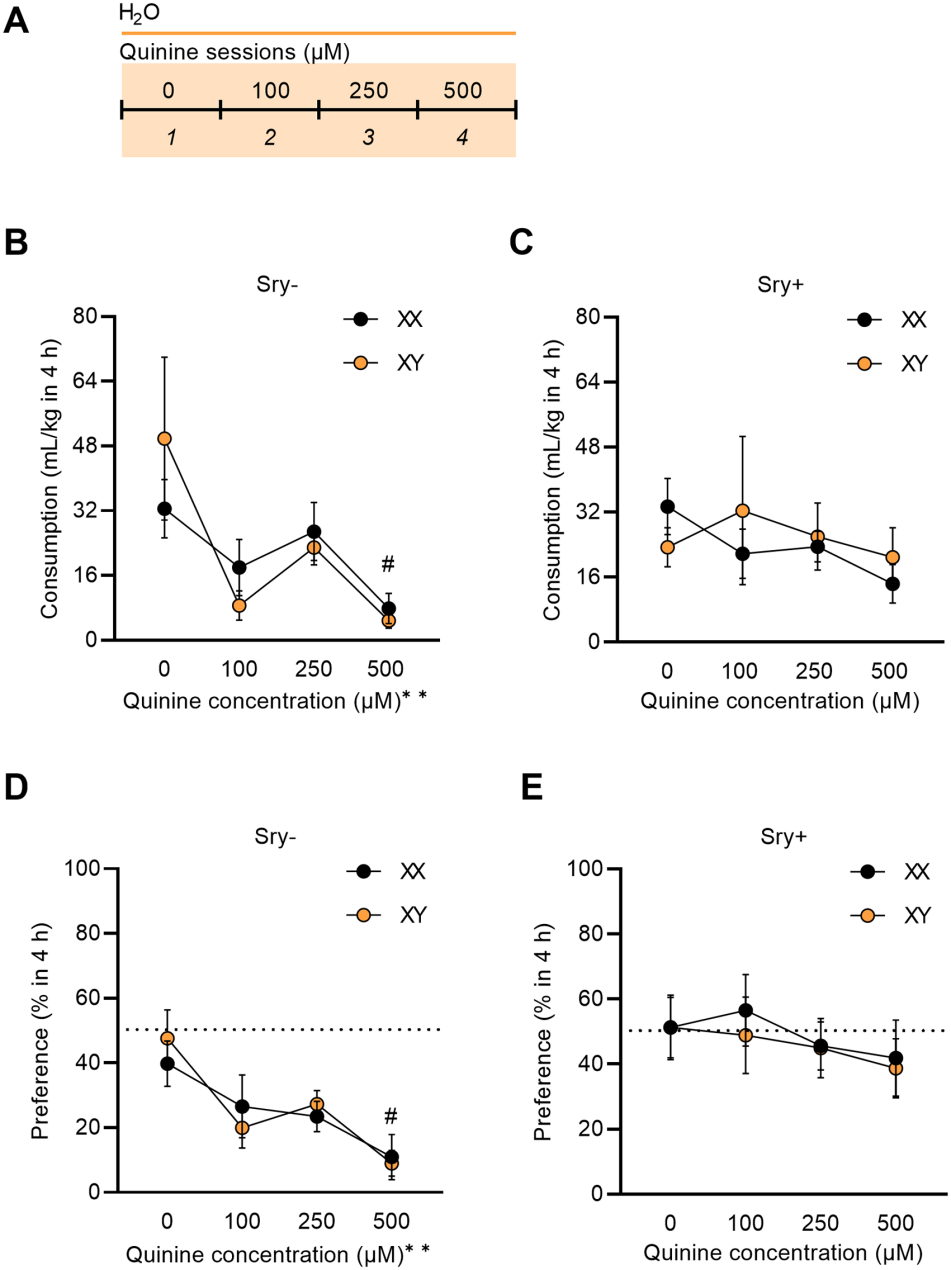
*Sry +* mice show insensitivity to quinine. **(A)** Mice (XX/*Sry –*: *n* = 14, XY/*Sry –*: *n* = 14, XX/*Sry +: n* = 14, and XY/*Sry +*: *n* = 14) had access to two bottles of water for one baseline session then one of the bottles was adulterated with increasing concentrations of quinine (100, 250, and 500 μM) across sessions 2–4. The bottles were alternated each session to control for side bias. **(B)** *Sry –* mice decrease their consumption of quinine + water across all concentrations of quinine. **(C)** *Sry +* mice do not show sensitivity to quinine at any concentration of quinine. **(D)** *Sry –* mice show less preference for water + quinine across all concentrations. **(E)** Preference is not altered when quinine is added to water in *Sry +* mice. All data expressed as mean ± standard error of the mean (SEM). ^#^*p* < 0.05 vs. 0 μM (Dunnett’s), ^**^p < 0.01 (main effect, Two–Way RM ANOVA).

Quinine sensitivity was assessed in a naive cohort of FCG mice (XX/*Sry–*: *n* = 14, XY/*Sry–*: *n* = 14, XX/*Sry+: n* = 14, and XY/*Sry+*: *n* = 14). Mice had access to two bottles of RO water for 4 h, similar to the home-cage EtOH drinking experiment outlined above. On sessions 2–4, quinine hemisulfate was added to the water in increasing concentrations (100, 250, and 500 μM; Figure 5A); as previously described in (24, 27–29, 37). Bottles were alternated daily to equate side bias.

### 2.10. Loss of righting reflex

To assess the sedative effects of EtOH in the FCG line, 39 EtOH-naïve mice (XX/*Sry–*: *n* = 9, XY/*Sry–*: *n* = 10, XX/*Sry+: n* = 10, and XY/*Sry+*: *n* = 10) were tested for the loss of righting reflex (LORR) (38). Mice were transferred to the experimental room 1 h before testing and were then weighed and given a 3.5 g/kg i.p. injection of 20% EtOH (*v*/*v*). A timer was started at the time of injection and the mice were placed in the supine position. When a mouse was no longer able to right itself onto all four paws, the latency to LORR was logged. When the mouse righted itself twice in 30 s, the time was logged as the LORR duration.

### 2.11. Blood EtOH concentration analysis

Immediately after a mouse regained the righting reflex, as described above, blood was taken using the tail clip technique (20). Blood samples were kept on ice and then were centrifuged using an accuSpinMicro17 microcentrifuge (Fisher Scientific) at 3,000 rpm for 8 min. Serum was pipetted into a sterile centrifuge tube and was then stored at −80°C until testing. An AM1 alcohol analyzer (Analox Technologies) was used to assess blood EtOH concentrations (BECs).

### 2.12. Data analysis

In the home-cage drinking experiments, consumption was calculated as (*Initial Bottle Weight* – *Post Bottle Weight*) – *Average of Dummy Bottles* and was expressed as grams of EtOH or mL of water per kilogram of body weight. To assess front-loading behavior for EtOH, each timepoint was normalized to match consumption in 30 min using the following calculations: 2-h timepoint as ((*2-h timepoint* – *30-min timepoint*)/3) and 4-h timepoint as ((*4-h timepoint* – *2-h timepoint*)/4). Preference was calculated as ((*Volume of EtOH*)/(*Volume of EtOH* + *Water Consumption*))*100. The 0 μM baseline consisted of an average of the last 5 EtOH drinking sessions (11–15) prior to quinine-exposure. Water consumption was averaged across drinking sessions. Initially, data were analyzed for all timepoints, 30 min, 2 h, and 4 h. Since the statistical trends were the same across all timepoints, only data from the 4 h timepoint are reported.

Consumption and preference for EtOH and water data were analyzed separately by gonad type since the mice with ovaries (*Sry–*) received an additional manipulation (vaginal lavage) to collect estrous cycle data and were run separately from mice with testes (*Sry+*). Data from bottles spilling or measurement error were excluded from all analyses. For the first 15 sessions of home-cage drinking, repeated measures (RM) Two-Way analysis of variance (ANOVA) or a Mixed Effects ANOVAs with the chromosome complement as the between-subjects factor and session as the within-subject factor were used. Mixed Effects ANOVA was used in the case of missing values as it gives the same results as a RM ANOVA and can be interpreted as such. Data for the quinine sessions were first analyzed at each concentration with session as the within-subjects factor and chromosome complement as the between-subjects factor. Since no interactions with session were observed, analyses of average consumption/preference are presented with quinine concentration as the within-subjects factor and chromosome complement as the between-subjects factor.

For estrous cycle data, a Mixed Effects ANOVA with estrous phase as the within-subjects factor and chromosome complement as the between-subjects factor was used. Mice were excluded if the phase of the cycle could not be determined for a particular quinine session.

For operant response experiments, data are expressed as the number of entries made into the active nose poke hole, inactive nose poke hole, or reward receptacle per session. For each mouse, responses were averaged over the last 3 sessions of each solution presented. Mice that did not make a minimum of 10 responses for the lowest concentration of EtOH presented (averaged over the final 3 sessions) were excluded from all analyses. Two data points are missing from the head entry data in the quinine experiment due to technical malfunction. For each experiment, data were analyzed using RM Three-Way ANOVA with concentration (EtOH or quinine) as the within-subject factor and chromosome complement and *Sry* as the between-subject factors. For the final test of responding with footshock, shock amplitude was used as the within-subjects factor.

For the EtOH sensitivity test, latency to LORR was measured as the time for the subject to sedate and lose the righting reflex. Duration of LORR was measured as how long it took the subject to right itself following LORR and was measured independently of LORR. Two-Way ANOVA with chromosome complement and *Sry* as the between-subject factors was used for the latency to LORR, duration of LORR, and BEC analyses.

For cases where sphericity was violated (*ε* < 0.75), the Greenhouse–Geisser correction was applied to the results of the ANOVA test. *Post hoc* tests and planned comparison’s corrected for multiple comparisons were used as appropriate (39–41). Specifically, a Holm Sidak’s test was used to compare groups and a Dunnett’s test was used to assess frontloading and quinine-resistant consumption/responding. All data were expressed as mean ± standard error of the mean and was analyzed using GraphPad Prism v. 9.0 (La Jolla, CA).

## 3. Results

### 3.1. Limited access home-cage EtOH drinking in the dark

#### 3.1.1. Sex chromosomes influence EtOH intake only in mice with testes

For *Sry–* mice, a RM Two Way ANOVA on consumption of 15% EtOH in 4 h found a main effect of session (*F*_(4.951, 128.735)_ = 4.858, *p* = 0.0004) but no main effect of chromosome complement (*F*_(1, 26)_ = 0.054, *p* = 0.818) and no interaction between these factors (*F*_(14, 364)_ = 0.585, *p* = 0.877; Figure 1B). Mice with XX and XY chromosomes in the *Sry–* group exhibited frontloading behavior; a RM Two Way ANOVA revealed a main effect of time point (*F*_(1.350_, _35.101)_ = 80.175, *p* < 0.0001) but no main effect of chromosome complement (*F*_(1, 26)_ = 0.102, *p* = 0.752) and no interaction between these factors (*F*_(2, 52)_ = 0.905, *p* = 0.411). A *post hoc* Dunnett’s test found that mice with the XX chromosome complement consumed less 15% EtOH during the 2 h (*p* = 0.0001) and 4 h (*p* = 0.00012) time points compared to the 30 min time point. Likewise, mice with the XY chromosome complement consumed less 15% EtOH during the 2 h (*p* < 0.0001) and 4 h (*p* < 0.0001) time points compared to the 30 min time point (Figure 1C).

For *Sry–* mice, those with the XY chromosome complement showed greater preference for 15% EtOH vs. water in 4 h. A Mixed Effects ANOVA identified main effects of chromosome complement (*F*_(1, 26)_ = 7.590, *p* = 0.011) and session (*F*_(8.560, 213.996)_ = 6.543, *p* < 0.0001) and an interaction between these factors (*F*_(14, 350)_ = 2.422, *p* = 0.003). A *post hoc* Holm Sidak’s test found that XY mice showed greater preference on sessions 3 (*p* = 0.040) and 15 (*p* = 0.025) vs. the mice with XX chromosomes (Figure 1D).

For *Sry +* mice, a Mixed Effects ANOVA on consumption of 15% EtOH in 4 h revealed main effects of chromosome complement (*F*_(1, 26)_ = 6.105, *p* = 0.020) and of session (*F*_(5.476, 141.985)_ = 5.987, *p* < 0.0001) and no interaction between these factors (*F*_(14, 363)_ = 0.993, *p* = 0.460; Figure 1E). Mice with XX and XY chromosomes in the *Sry +* group displayed frontloading behavior. A RM Two Way ANOVA found a main effect of time point (*F*_(1.076, 27.974)_ = 114.717, p < 0.0001) but no main effect of chromosome complement (*F*_(1, 26)_ = 3.117, *p* = 0.089) and no interaction between these factors (*F*_(2, 52)_ = 0.392, *p* = 0.678). A *post hoc* Dunnett’s test found that mice of both genotypes consumed less 15% EtOH during the 2 h (*p* < 0.0001) and 4 h (*p* < 0.0001) time points compared to the 30 min time point (Figure 1F).

For *Sry +* mice, those with the XY chromosome complement showed greater preference for 15% EtOH vs. water in 4 h. A RM Two Way ANOVA revealed main effects of chromosome complement (*F*_(1, 26)_ = 5.321, *p* = 0.029) and session (*F*_(6.533, 169.876)_ = 2.738, *p* = 0.012) and no interaction between these factors (*F*_(14, 364)_ = 0.956, *p* = 0.499; Figure 1G).

#### 3.1.2. Sex chromosomes influence water intake only in mice with ovaries

For *Sry–* mice, a Mixed Effects ANOVA on consumption of water in 4 h found main effects of chromosome complement (*F*_(1, 26)_ = 4.900, *p* = 0.036) and session (*F*_(5.317, 132.927)_ = 4.695, *p* = 0.0004) but no interaction between these factors (*F*_(14, 350)_ = 1.408, *p* = 0.146; Table 1).

**Table 1:** Four core genotypes (FCG) water consumption in 4 h across session.

Genotype	Session^*^															AVG (mL/kg)
	1	2	3	4	5	6	7	8	9	10	11	12	13	14	15	
XX/Sry–^*^	24.908 ± 4.551	30.942 ± 6.874	28.416 ± 5.434	19.161 ± 3.958	25.322 ± 3.027	6.966 ± 2.383	20.991 ± 2.463	11.599 ± 3.942	15.569 ± 2.935	6.752 ± 8.537	21.752 ± 3.976	14.911 ± 6.198	15.448 ± 4.406	16.427 ± 8.378	12.282 ± 4.308	17.670 ± 2.613
XY/*Sry–*	26.318 ± 10.338	23.830 ± 8.817	6.637 ± 2.194	16.559 ± 3.979	9.662 ± 3.282	10.514 ± 4.697	11.533 ± 2.929	12.091 ± 4.319	11.735 ± 4.425	4.730 ± 2.982	12.096 ± 3.547	4.625 ± 2.531	3.567 ± 1.354	7.069 ± 3.019	0.525 ± 0.433	10.340 ± 2.104
XX/*Sry+*	18.787 ± 4.941	14.770 ± 3.059	15.569 ± 3.635	15.471 ± 2.477	9.584 ± 2.601	19.281 ± 6.775	24.944 ± 6.046	24.403 ± 7.648	18.392 ± 4.680	20.086 ± 5.663	14.820 ± 5.613	16.026 ± 7.404	19.101 ± 3.833	18.676 ± 8.850	14.428 ± 4.961	17.622 ± 3.232
XY/*Sry+*	12.284 ± 4.737	16.669 ± 4.143	15.291 ± 4.528	16.369 ± 3.737	11.068 ± 3.737	10.819 ± 3.059	16.347 ± 2.238	8.318 ± 3.140	13.099 ± 3.571	9.436 ± 2.918	8.091 ± 3.025	14.065 ± 4.697	5.864 ± 2.117	14.904 ± 4.419	12.671 ± 5.235	12.354 ± 2.047

Main effect of session and chromosome complement for Sry – mice. ^*^p < 0.05.

XX chromosomes promote water intake in *Sry –* mice (XX/*Sry –*: *n* = 14, XY/*Sry –*: *n* = 14) across drinking session and on average. No effect of chromosomes in *Sry +* mice (XX/*Sry +: n* = 14, and XY/*Sry +*: *n* = 14) were observed. Comparisons were made between chromosomes for each gonad type. Data expressed as averages ± standard error of the mean (SEM). ^*^p < 0.05 (2-Way ANOVA).

For *Sry +* mice, A RM Two Way ANOVA on consumption of water in 4 h identified no main effects of chromosome complement (*F*_(1, 26)_ = 1.896, *p* = 0.180) or session (*F*_(5.282, 137.337)_ = 0.716) and no interaction between these factors (F_(14, 364)_ = 0.914, *p* = 0.544; Table 1).

#### 3.1.3. Sex chromosomes influence aversion-resistant home-cage EtOH intake

For *Sry–* mice, when consumption of quinine +15% EtOH was averaged across the five sessions per concentration, a RM Two Way ANOVA identified a main effect of quinine concentration (*F*_(1.989, 51.717)_ = 10.754, *p* = 0.001) and an interaction between quinine concentration X chromosome complement (*F*_(3, 78)_ = 6.867, p = 0.0004) but no main effect of chromosome complement (*F*_(1, 26)_ = 2.485, *p* = 0.127). A *post hoc* Dunnett’s test revealed that mice with the XX chromosome complement reduced consumption of 15% EtOH adulterated with 250 μM (*p* = 0.025) and 500 μM (*p* = 0.0006) quinine. When comparing between groups, a *post hoc* Holm Sidak’s test revealed that mice with the XX chromosome complement consumed less of 500 μM quinine vs. mice with the XY chromosome complement (*p* = 0.011; Figure 2A).

For *Sry +* mice, when consumption of quinine +15% EtOH was averaged across the 5 sessions per concentration, a RM Two Way ANOVA found main effects of chromosome complement (*F*_(1, 26)_ = 8.235, *p* = 0.008) and quinine concentration (*F*_(2.258, 58.720)_ = 5.210, *p* = 0.006) but no interaction between these factors (*F*_(3, 78)_ = 1.555, *p* = 0.207). A *post hoc* Dunnett’s test showed that mice with the XY chromosome complement reduced consumption of 15% EtOH adulterated with 500 μM (*p* = 0.016) quinine. When comparing between groups, a *post hoc* Holm Sidak’s test found that mice with the XX chromosome complement consumed less EtOH with 0 μM (*p* = 0.028), 100 μM (*p* = 0.028), and 250 μM (*p* = 0.030) quinine vs. mice with the XY chromosome complement (Figure 2B).

For *Sry–* mice, when preference for quinine + EtOH was averaged across the 5 sessions per concentration, a RM Two Way ANOVA found main effects of chromosome complement (*F*_(1, 26)_ = 14.816, *p* = 0.0007) and quinine concentration (*F*_(2.273, 59.093)_ = 18.048, *p* < 0.0001) but no interaction between these factors (*F*_(3, 78)_ = 1.970, *p* = 0.125). A *post hoc* Dunnett’s test revealed that preference for EtOH + quinine was suppressed for the 250 and 500 μM quinine concentration for mice with the XX (*p* = 0.021, *p* < 0.0001, respectively) and XY (*p* = 0.029, *p* = 0.023, respectively) chromosome complements. When comparing between groups, a *post hoc* Holm Sidak’s test identified that mice with the XX chromosome complement consumed less EtOH with 0 μM (*p* = 0.007), 100 μM (p = 0.007), 250 μM (*p* = 0.020), and 500 μM (p = 0.007) quinine vs. mice with the XY chromosome complement (Figure 2C).

For *Sry +* mice, when preference for quinine + EtOH was averaged across the 5 sessions per concentration, a RM Two Way ANOVA identified no main effects of chromosome complement (*F*_(1, 26)_ = 3.346, *p* = 0.079) or quinine concentration (*F*_(2.368, 61.557)_ = 0.651, *p* = 0.550) and no interaction between these factors (*F*_(3, 78)_ = 1.810, *p* = 0.152; Figure 2D).

For water consumption in *Sry–* mice when consumption data was averaged across the 5 sessions per quinine concentration, a RM Two Way ANOVA found a main effect of quinine concentration (*F*_(1.7121, 44.514)_ = 11.433, *p* = 0.0002) and chromosome complement (*F*_(1, 26)_ = 6.873, *p* = 0.014) and no interaction between these factors (F_(3, 78)_ = 0.589, *p* = 0.624). A *post hoc* Dunnett’s test identified that water consumption increased when 250 μM (*p* = 0.003) and 500 μM (p = 0.014) quinine was present in EtOH for mice with the XX chromosome complement and was higher only when 500 μM was present in EtOH for the mice with the XY chromosome complement (*p* = 0.011; Table 2).

**Table 2:** FCG water consumption during quinine sessions.

Genotype	100 μM					AVG (mL/kg)	250 μM					AVG (mL/kg)	500 μM					AVG (mL/kg)
	1	2	3	4	5		1	2	3	4	5		1	2	3	4	5	
XX/Sry–^*^	8.338 ± 3.117	23.013 ± 10.916	25.081 ± 4.501	30.230 ± 9.527	12.649 ± 4.005	20.462 ± 2.806	20.077 ± 4.125	45.687 ± 23.387	18.364 ± 4.792	31.960 ± 6.421	32.655 ± 9.370	29.749 ± 6.115	26.863 ± 10.85	46.144 ± 17.196	26.344 ± 7.023	27.129 ± 6.084	39.360 ± 11.557	33.168 ± 8.109
XY/*Sry–*	5.355 ± 2.019	9.328 ± 3.983	14.647 ± 4.299	6.807 ± 2.725	5.064 ± 1.905	8.240 ± 2.221	12.608 ± 4.810	8.832 ± 2.678	14.503 ± 5.816	10.196 ± 2.491	17.161 ± 3.933	12.660 ± 2.664	8.311 ± 2.661	18.769 ± 4.478	11.823 ± 4.073	32.866 ± 8.690	16.394 ± 6.317	17.633 ± 3.607
XX/*Sry+*	11.147 ± 3.765	11.115 ± 3.510	15.718 ± 4.379	12.155 ± 3.285	25.243 ± 33.297	15.076 ± 3.913	10.779 ± 3.175	14.328 ± 3.767	17.592 ± 4.376	12.065 ± 4.355	10.135 ± 2.608	12.980 ± 2.381	5.885 ± 2.462	13.945 ± 3.522	16.616 ± 2.970	13.205 ± 6.200	10.784 ± 3.461	12.087 ± 2.360
XY/*Sry+*	20.526 ± 13.792	5.456 ± 2.035	9.275 ± 3.179	10.907 ± 3.108	15.639 ± 4.232	12.360 ± 3.292	12.348 ± 3.849	15.478 ± 5.441	21.352 ± 6.476	8.549 ± 2.484	17.025 ± 4.485	14.950 ± 2.903	20.832 ± 5.950	16.261 ± 5.241	20.601 ± 3.777	14.284 ± 4.220	8.028 ± 2.334	16.001 ± 2.666

Main effect of chromosome complement for the *Sry–* mice during the 100 and 250 μM sessions. ^*^p < 0.05.

Water consumption during the 100, 250, and 500 μM quinine + EtOH sessions. For *Sry–* mice (XX/*Sry–*: *n* = 14, XY/*Sry–*: *n* = 14) with the XX chromosome complement consumed more water during the 100 and 250 μM sessions. For *Sry +* mice (XX/*Sry+: n* = 14, and XY/*Sry+*: *n* = 14), no differences were observed between groups. Data expressed as averages ± standard error of the mean (SEM). ^*^p < 0.05 (Two-Way ANOVA).

For water consumption in *Sry +* mice when consumption data was averaged across the 5 sessions per quinine concentration, a RM Two Way ANOVA revealed no main effects of chromosome complement (*F*_(1, 26)_ = 0.029, *p* = 0.867) or quinine concentration (*F*_(1.865, 48.495_) = 0.007, *p* = 0.991) and no interaction between these factors (*F*_(3, 78)_ = 1.570, *p* = 0.203; Table 2).

#### 3.1.4. The estrous cycle does not influence aversion resistant EtOH drinking, preference, or water intake

A Mixed ANOVA on consumption for 100 μM quinine + EtOH found no main effects of estrous phase (*F*_(2.686, 61.783)_ = 0.655, *p* = 0.567) or chromosome complement (*F*_(1, 26)_ = 0.687, *p* = 0.415) and no interaction between these factors (*F*_(3, 69)_ = 0.174, *p* = 0.914). A Mixed ANOVA on consumption for 250 μM quinine + EtOH revealed no main effects of estrous phase (*F*_(2.420, 51.625)_ = 0.152, *p* = 0.895) or chromosome complement (*F*_(1, 25)_ = 4.189, *p* = 0.051) and no interaction between these factors (*F*_(3, 64)_ = 0.891, *p* = 0.451). A Mixed ANOVA on consumption for 500 μM quinine + EtOH identified a main effect of chromosome complement (*F*_(1, 24)_ = 15.046, *p* = 0.0007) but no main effect of estrous phase (*F*_(2.554, 51.072)_ = 1.717, *p* = 0.182) and no interaction between these factors (*F*_(3, 60)_ = 1.618, *p* = 0.195; Figure 2F). Mice in both the XX and XY groups spent 1 day in each cycle phase.

A Mixed ANOVA on preference for 100 μM quinine + EtOH found a main effect of chromosome complement (*F*_(1, 26)_ = 9.553, *p* = 0.005) but no main effect of estrous phase (*F*_(2.241, 51.539)_ = 0.640, *p* = 0.548) and no interaction between these factors (F_(3, 69)_ = 0.728, *p* = 0.539). A Mixed ANOVA on preference for 250 μM quinine + EtOH revealed a main effect of chromosome complement (*F*_(1, 25)_ = 15.882, *p* = 0.0005) but no main effect of estrous phase (*F*_(2.549, 55.223)_ = 0.194, *p* = 0.872) and no interaction between these factors (*F*_(3, 65)_ = 1.171, *p* = 0.328). A Mixed ANOVA on preference for 500 μM quinine + EtOH identified a main effect of chromosome complement (*F*_(1, 24)_ = 10.053, *p* = 0.004) but no main effect of estrous phase (*F*_(2.867, 57.330)_ = 1.730, *p* = 0.173) and no interaction between these factors (*F*_(3, 60)_ = 0.517, *p* = 0.672; Figure 2G). Mice in both the XX and XY groups spent 1 day in each cycle phase.

A Mixed ANOVA on water consumption during the 100 μM quinine + EtOH session found a main effect of chromosome complement (*F*_(1, 95)_ = 8.679, *p* = 0.004) but no main effect of estrous phase (*F*_(2.248, 71.184)_ = 0.302, *p* = 0.766) and no interaction between these factors (*F*_(3, 95)_ = 1.074, *p* = 0.364). A Mixed ANOVA on water consumption during the 250 μM quinine + EtOH session revealed a main effect of chromosome complement (*F*_(1, 25)_ = 10.617, *p* = 0.003) but no main effect of estrous phase (*F*_(2.561, 54.626)_ = 0.781, *p* = 0.492) and no interaction between these factors (*F*_(3, 64)_ = 1.628, *p* = 0.192). A Mixed ANOVA on water consumption during the 500 μM quinine + EtOH session identified no main effects of chromosome complement (F_(1, 24)_ = 3.291, *p* = 0.082) and estrous phase (*F*_(1.916, 38.328)_ = 0.558, *p* = 0.570) and no interaction between these factors (*F*_(3, 60)_ = 0.597, *p* = 0.619; Table 3). Mice in both the XX and XY groups spent 1 day in each cycle phase.

**Table 3:** The estrous cycle does not influence water consumption during quinine sessions in mice with ovaries.

	Genotype	100 μM		250 μM		500 μM	
		XX/Sry–^**^	XY/*Sry–*	XX/Sry–^**^	XY/*Sry–*	XX/*Sry–*	XY/*Sry–*
Phase	Estrus	16.368±9.496	13.521±5.617	29.387±11.040	4.3225±1.920	35.289±17.420	17.173±6.772
	Proestrus	23.688±4.980	10.525±4.497	37.712±7.541	13.303±4.697	39.476±9.596	16.671±4.668
	Metestrus	20.379±9.496	7.708±2.638	18.769±6.006	16.941±5.079	27.932±7.556	17.589±5.352
	Diestrus	32.978±12.066	6.512±2.301	27.026±5.995	11.429±3.200	22.286±5.097	14.354±5.476

Main effect of chromosome complement during the 100 and 250 μM sessions ^**^p < 0.01.

Water consumption during the 100, 250, and 500 μM quinine + EtOH sessions did not vary by estrous phase. Mice with the XX chromosomes complement consume more water during the 100 and 250 μM sessions. Data expressed as averages ± standard error of the mean (SEM). ^**^p < 0.01 (2-Way ANOVA).

### 3.2. Operant responding for EtOH

#### 3.2.1. Sex chromosomes influence concentration-dependent responding for EtOH in *Sry* + mice

In the cohort of mice tested with escalating EtOH concentrations, five mice did not reach the criterion of an average 10 responses for the 5% concentration of EtOH (XX/*Sry–*: *n* = 1, XX/*Sry+: n* = 1, and XY/*Sry+*: *n* = 3) and were excluded from the analyses. Responding on the inactive nose poke hole was low for all groups during response training and testing (< 10 responses), indicating that mice successfully learned to respond for reward.

All four genotypes responded for the 10% sucrose +10% EtOH and 5% sucrose +5% EtOH solutions (Figure 3B). A RM Three Way ANOVA conducted on responses during response training revealed no main effects of concentration (*F*_(1,31)_ = 1.940, *p* = 0.174), *Sry* (*F*_(1,31)_ = 0.395, *p* = 0.534) or chromosome complement (*F*_(1,31)_ = 0.096, *p* = 0.759). There were also no significant interactions, including between concentration, *Sry*, and chromosome complement (all *p* > 0.336). When examining head entries into the reward receptacle (Figure 3C), the interaction of concentration and chromosomes neared significance (*F*_(1,31)_ = 3.899, *p* = 0.057), but other no significant main effects or interactions were observed (all *p* > 0.505).

When examining responding for the escalating EtOH concentrations, a RM Three Way ANOVA showed a main effect of EtOH concentration (*F*_(2.768, 85.80)_ = 23.230, *p* < 0.0001; Figure 3D). There was no main effect of chromosome complement (*F*_(1,31)_ = 0.013, *p* = 0.910) but the main effect of *Sry* approached the threshold of significance (*F*_(1,31)_ = 3.988, *p* = 0.055). A significant three-way interaction between all of these factors was also found (*F*_(3,93)_ = 3.691, *p* = 0.015). To explore this interaction, RM Two Way ANOVAs were performed separately on data from *Sry–* and *Sry* + mice. For *Sry–* mice, there was a significant main effect of concentration (*F*_(2.183,37.120)_ = 17.930, *p* < 0.0001) but no main effect of chromosomes (*F*_(1,17)_ = 0.278, *p* = 0.605) or interaction between the two factors (*F*_(3,51)_ = 1.397, *p* = 0.254). *Post hoc* Holm Sidak’s tests comparing responding at each concentration for XX/*Sry–* mice found that responses were significantly lower for 10% (*p* = 0.020), 15% (*p* = 0.012) and 20% (*p* = 0.012) vs. 5% EtOH. Comparisons between all other concentrations were not significant (all *p* > 0.234) and there were no differences between XX and XY mice at any concentration (all *p* > 0.689). *Post hoc* Holm Sidak’s tests comparing responding at each concentration for XY/*Sry–* mice found that responses were significantly lower for 15% (*p* = 0.016) and 20% (*p* = 0.0009) vs. 5% EtOH. Comparisons between all other concentrations were not significant (all *p* > 0.290) and there were no differences between XX and XY mice at any concentration (all *p* > 0.378).

For *Sry* + mice, there was a significant main effect of concentration (*F*_(3,42)_ = 7.900, *p* = 0.0003) but no main effect of chromosomes (*F*_(1,14)_ = 0.359, *p* = 0.559) and the interaction between the two factors approached the threshold of significance (*F*_(3,42)_ = 2.726, *p* = 0.056). *Post hoc* Holm Sidak’s tests comparing responding at each concentration for XX/*Sry* + mice were not significant (all *p* > 0.688). *Post hoc* Holm Sidak’s tests comparing responding at each concentration for XY/*Sry* + mice found that responses were significantly lower for 10% (*p* = 0.007), 15% (*p* = 0.0002) and 20% (*p* = 0.0005) vs. 5% EtOH. Comparisons between all other concentrations were not significant (all *p* > 0.543).

*Sry* + mice also made more entries into the reward receptacle compared to *Sry–* mice (Figure 3E). A RM Three Way ANOVA revealed a significant main effect of Sry (*F*_(1,31)_ = 6.758, *p* = 0.0142). There was no effect of chromosomes or concentration and no interactions between any of the factors (all *p* > 0.127).

#### 3.2.2. Four core genotypes mice do not reduce responding for quinine-adulterated EtOH

For mice tested for aversion-resistant operant responding, 18 mice did not reach the criterion of an average 10 responses for the 10% concentration of EtOH (XX/*Sry–*: *n* = 2, XY/*Sry–*: *n* = 9, XX/*Sry+: n* = 2, and XY/*Sry+*: *n* = 5) and were excluded from the analyses. Exclusion of these mice resulted in a total n = 40 (XX/*Sry–*: *n* = 10, XY/*Sry–*: *n* = 10, XX/*Sry+: n* = 11, and XY/*Sry+*: *n* = 9). Responding on the inactive nose poke hole was low for all groups during response training and testing (< 7 responses), indicating that mice successfully learned to respond for reward.

All four genotypes decreased responding as the concentration of sucrose dissolved in 10% EtOH decreased from 10 to 5%. A RM Three Way ANOVA revealed a main effect of concentration on responses during response training (*F*_(1,35)_ = 19.60, *p* < 0.0001) but no effect of *Sry* (*F*_(1,35)_ = 0.182, *p* = 0.672) or chromosome complement (*F*_(1,35)_ = 0.003, *p* = 0.959; Figure 4B). There were no significant interactions between any of the factors (all *p* > 0.199). XX mice made more head entries into the reward receptacle than XY mice (Figure 4C), as indicated by a significant main effect of chromosomes (*F*_(1,33)_ = 4.172, *p* = 0.049). A significant main effect of concentration was also observed (*F*_(1,33)_ = 15.560, *p* = 0.0004) but no main effect of Sry. There were no significant interactions between any of the factors (all *p* > 0.133).

The RM Three-Way ANOVA of the number of responses made by mice during the quinine sessions revealed no significant main effect of concentration (*F*_(2.073,72.540)_ = 2.173, *p* = 0.119; Figure 4D), main effect of *Sry* gene (F_(1,35)_ = 1.020, *p* = 0.319), or chromosome complement (F_(1,35)_ = 0.552, *p* = 0.462). The interaction of *Sry* and chromosomes neared the threshold of significance (F_(1,35)_ = 3.635, *p* = 0.065), but there were no other significant interactions between the factors (all *p* > 0.225). For head entries into the reward receptacle (Figure 4E), the main effect of chromosomes (*F*_(1,31)_ = 3.923, *p* = 0.057) and the interaction of *Sry* and concentration (*F*_(3,93)_ = 2.698, *p* = 0.050) neared the threshold for significance. No other significant main effects or interactions were observed (all *p* > 0.1250).

#### 3.2.3. Four core genotypes reduce responding for EtOH when paired with a footshock

Because quinine was ineffective in reducing responding for EtOH, a subset of mice were tested with a 0.35 mA footshock on one session to obtain preliminary data. We have previously found sex differences in footshock-resistant responding for EtOH in C57BL/6 J mice using the same procedure employed here (39). Footshock reduced responses in all mice, regardless of genotype, when compared to responding for 10% EtOH with 0 μM quinine. When assessing the responses during the shock session, a RM Three Way ANOVA identified a main effect of footshock (*F*_(1,9)_ = 25.18, *p* = 0.0007). There were no main effects of *Sry* (F_(1,9)_ = 0.335, *p* = 0.577) or chromosome complement (F_(1,9)_ = 0.347, *p* = 0.571). All interactions were not significant (all *p* > 0.466), although the interaction between *Sry* and chromosome complement approached the threshold of significance (F_(1,9)_ = 4.664, *p* = 0.059; Figure 4F). When assessing head entries into the reward receptacle with a RM Three-Way ANOVA, footshock was found to reduce head entries (Figure 4G), as evidenced by a significant main effect of footshock (*F*_(1,9)_ = 21.26, *p* = 0.0013). There were no other significant main effects or interactions (all *p* > 0.110).

### 3.3. *Sry +* mice show insensitivity to quinine

To assess quinine sensitivity, naïve FCG mice underwent a quinine sensitivity test where escalating concentrations of quinine were added to water. A RM Three Way ANOVA on consumption found a main effect of quinine concentration (*F*_(1.992, 103.605)_ = 6.061, *p* = 0.003) and no main effect of *Sry* (*F*_(1, 52)_ = 0.305, *p* = 0.583) or chromosome complement (F_(1, 52)_ = 0.0580, *p* = 0.811). The interaction between *Sry* and quinine concentration almost met the threshold of significance (*F*_(3, 156)_ = 2.491, *p* = 0.062). All other interactions were not significant (all *p* > 0.1559). To further explore these data, for *Sry–* mice, when assessing consumption of water adulterated with quinine in 4 h, a RM Two Way ANOVA found a main effect of quinine concentration (*F*_(1.450, 37.71)_ = 6.929, *p* = 0.006) but no main effect of chromosome complement (*F*_(1, 26)_ = 0.001, *p* = 0.973) and no interaction between these factors (*F*_(3, 78)_ = 1.029, *p* = 0.385). A *post hoc* Dunnett’s test found that both XX and XY mice in the *Sry–* group decreased consumption for 500 μM quinine compared to 0 μM quinine (*p* = 0.034; Figure 5B). For *Sry +* mice, when assessing consumption of water adulterated with quinine in 4 h, a RM Two Way ANOVA found no main effects of quinine concentration (*F*_(1.631, 42.405)_ = 0.8755, *p* = 0.404) or chromosome complement (*F*_(1, 26)_ = 0.082, *p* = 0.777) and no interaction between these factors (*F*_(3, 78)_ = 0.763, *p* = 0.518; Figure 5C).

A RM Three Way ANOVA on preference found a main effect of quinine concentration (*F*_(2.835, 147.423)_ = 5.531, *p* = 0.002) and *Sry* (F_(1, 52)_ = 17.817, *p* < 0.0001) and no main effect of chromosome complement (F_(1, 52)_ = 0.040, *p* = 0.841). All interactions were not significant (all *p* > 0.1425). To further explore these data, for *Sry–* mice a RM Two Way ANOVA revealed a main effect of quinine concentration (*F*_(2.438, 63.395)_ = 8.196, *p* = 0.0003) but no main effect of chromosome complement (F_(1, 26)_ = 0.031, *p* = 0.862) and no interaction between these factors (F_(3, 78)_ = 0.429, *p* = 0.733). A *post hoc* Dunnett’s test identified that mice with the XX (p = 0.034) and XY (p = 0.002) chromosome complement in the *Sry–* group showed a decline in preference for the 500 μM quinine compared to 0 μM quinine (Figure 5D). For *Sry +* mice a RM Two Way ANOVA identified no main effects of quinine concentration (*F*_(2.575, 66.949)_ = 0.871, *p* = 0.447) or chromosome complement (F_(1, 26)_ = 0.094, *p* = 0.762) and no interaction between these factors (F_(3, 78)_ = 0.0803, *p* = 0.971; Figure 5E).

### 3.4. The sedative effects of EtOH does not differ by genotype in FCG mice

When evaluating latency to LORR, a Two Way ANOVA found no main effects of *Sry* (*F*_(1, 35)_ = 1.481, *p* = 0.232) or chromosome complement (F_(1, 35)_ = 2.036, *p* = 0.163) and no interaction between these factors (F_(1, 35)_ = 0.033, *p* = 0.858). For duration of LORR, a Two Way ANOVA identified no main effects of *Sry* (F_(1, 35)_ = 0.636, *p* = 0.431) or chromosome complement (F_(1, 35)_ = 1.330, *p* = 0.267) and no interaction between these factors (F_(1, 35)_ = 0.627, *p* = 0.434; Table 1).

Blood EtOH concentrations measured after mice regained the righting reflex did not differ among FCG mice. A Two Way ANOVA revealed no main effects of *Sry* (*F*_(1, 36)_ = 0.228, *p* = 0.636) or chromosome complement (F_(1, 36)_ = 2.544, *p* = 0.119) and no interaction between these factors (F_(1, 36)_ = 0.730, *p* = 0.399; Table 4).

**Table 4:** Sedative effects of EtOH on FCG mice.

Genotype	Latency to LORR (min)	Duration of LORR (min)	BECs at RORR (mg/dL)
XX/*Sry–*	4.044±2.725	56.602±7.692	350.615±25.425
XY/*Sry–*	6.525±1.820	76.180±10.968	338.210±13.301
XX/*Sry+*	1.261±0.155	58.551±7.574	372.930±11.006
XY/*Sry+*	4.462±2.392	61.818±9.173	331.895±13.340

FCG mice (XX/*Sry–*: *n* = 9, XY/*Sry–*: *n* = 10, XX/*Sry+: n* = 10, and XY/*Sry+*: *n* = 10) performed similarly in a loss of righting reflex test. No differences in blood ethanol concentration (BEC) were observed. Data expressed as averages ± standard error of the mean (SEM).

## 4. Discussion

The major findings of this study are that sex chromosome complement influences binge-like EtOH drinking, aversion-resistant drinking, and concentration-dependent responding for EtOH in the operant chamber. Considered alongside previous findings demonstrating a role for sex chromosomes in habitual responding for EtOH (19) and EtOH consumption, preference, and relapse-like behavior in a continuous access drinking paradigm (20), these results reveal an important role for chromosomal sex in multiple alcohol drinking behaviors.

### 4.1. Sex chromosome influences on binge-like drinking

The role of sex chromosomes in binge-like drinking was assessed separately in *Sry–* and *Sry* + mice using a two-bottle choice DID paradigm. For the *Sry–* mice, no chromosomal influences were observed across the 15 drinking sessions and drinking increased across sessions. Frontloading behavior did not differ between the genotypes. For the *Sry +* group, the mice with XX chromosomes consumed less 15% EtOH across the 15 drinking sessions and drinking varied across session for both groups, but no differences in frontloading behavior were observed. These results differ from previous studies of EtOH consumption in the FCG mice. In a study using FCG mice drinking 10% EtOH in a single 30-min session, no effect of sex chromosomes was observed (19). Also at odds with the current results is our previous report of greater consumption of 20% EtOH under continuous access conditions in XX mice regardless of gonadal sex (20).

When assessing preference for 15% EtOH vs. water, XY mice had greater preference across sessions in mice of both gonad type. In the *Sry–* mice, this effect is due to lower water consumption in the XY mice, as EtOH consumption did not differ by chromosomal sex. For the *Sry +* group, no differences in water consumption were observed between the genotypes, therefore preference differences can be explained by XY/*Sry +* mice drinking more 15% EtOH across sessions than their XX counterparts. Although differences in water consumption occurred independently of differences in EtOH intake in the current study, we have previously observed that gonadal sex increases consumption of both fluids in mice with ovaries (20, 42). This observation raises the question of whether commonly observed sex differences in EtOH drinking might sometimes be the result of more general increases in fluid consumption in female animals. Considering these effects, we think it is paramount for future investigations of sex differences in alcohol drinking behaviors to include appropriate controls for nonspecific effects on fluid consumption.

As noted above, both the consumption and preference findings are inconsistent with our prior work demonstrating greater consumption and preference in XX mice (20). A major difference between that study and the current one is the drinking schedule employed. Here, we studied binge-like drinking using a limited-access DID task while our previous study examined consumption during continuous access. Because the DID promotes greater and more rapid consumption than continuous access, this model produces BECs that are more pharmacologically relevant than standard continuous exposure models (43). Intermittent exposure to drugs of abuse is also thought to produce neuroadaptations that are critical for the development of addictive behavior, including in rodent models of alcohol drinking (44–49). Thus, the opposing results in the FCG mice highlight the fact that different EtOH drinking paradigms likely tap into separate but overlapping neurobiological mechanisms, such that they can be regulated by the same manipulation but in different directions. Additional studies with other exposure regimens and drugs of abuse may further clarify this point.

For the present experiment we ran our *Sry–* and *Sry +* groups separately and, therefore, could not statistically compare the differences between these groups based on gonad type. It is therefore unclear if there were any gonadal effects on EtOH or water consumption and preference in the DID paradigm, as have been previously observed using other drinking tasks (19, 20, 50–52). However, the finding that sex chromosome complement influenced binge-like consumption in *Sry* + mice only suggests that chromosomal sex likely interacts with gonadal status to control this behavior.

### 4.2. Sex chromosome influences on aversion-resistant intake

Chromosomal sex also influenced quinine-resistant consumption of 15% EtOH in the DID paradigm, although a major caveat of this study is that *Sry +* mice demonstrated insensitivity to quinine when it was presented in water. It is possible that *Sry +* mice are innately insensitive to the concentrations of quinine used here, as has been observed in lines of mice selectively bred for alcohol preference (53), and that higher concentrations are necessary to reduce drinking in these animals. Thus, although it may appear that XX/*Sry* + mice were more aversion-resistant than XY/*Sry* + mice, this result should be interpreted with caution. Because quinine sensitivity was preserved in *Sry–* mice, the finding of increased aversion-resistance in mice with the XY chromosome complement (vs. XX/*Sry–* mice), can be made with more confidence. These results agree with prior findings demonstrating that XY mice developed habitual responding for EtOH while XX mice remained sensitive to outcome devaluation (19). The results seen here in FCG mice also align with previous studies of quinine-resistant binge-like EtOH consumption in male and female C57BL/6 J mice (26, 27). These studies have shown that females are aversion-resistant at the 100 μM concentration but not the 250 μM or 500 μM quinine concentrations, similar to XX/*Sry–* mice in the current study. In contrast, XY/*Sry–* mice were aversion-resistant at all quinine concentrations.

Although consumption remained aversion-resistant in XY/*Sry–* mice, preference for quinine + EtOH decreased in both *Sry–* groups. These results with preference may be due to water consumption as average intake of water increased from 8.240 ± 2.221 ml/kg (100 μM) to 17.633 ± 3.607 ml/kg (500 μM) in the XY mice. Therefore, it seems that mice with the XY chromosome complement consumed more fluids only when quinine was added to the EtOH solution.

Although the estrous cycle has not been found to greatly influence most EtOH drinking behaviors (4, 54), we thought it was important to determine whether this was true in the FCG mice. In FCG mice, others have found that XX and XY mice with ovaries spent the same number of days in each cycle phase (55), which suggests that ovarian function is similar between these two groups. Here we found no differences in EtOH consumption, preference for quinine + EtOH, or water consumption at any quinine concentration across the different phases of the estrous cycle. In addition, the mice spent 1 day in each phase of the cycle. These results suggest that the day-to-day fluctuation of hormones does not influence aversion-resistant drinking in FCG mice with ovaries. This finding is consistent with the literature where others have not seen an influence of the estrous cycle on binge-like drinking in a DID paradigm (10) or quinine-resistant drinking in a continuous access paradigm (23). Although we did monitor the estrous cycle during aversion-resistant sessions, we did not monitor it during any of the initial DID drinking sessions. Therefore, it is unknown if there are any cycling differences between these two groups following EtOH exposure alone.

### 4.3. Sex chromosome influences on operant responding for EtOH

To complement the DID paradigm, FCG mice were also trained to respond for EtOH in an operant chamber. When testing mice with escalating concentrations of EtOH, there were no effect of *Sry* or sex chromosome complement, although the main effect of *Sry* approached the threshold of significance (*p* = 0.055). Additionally, XX/*Sry–*, XY/*Sry–*, and XY/*Sry* + mice reduced responding when the concentration of EtOH delivered was increased. Only XX/*Sry* + mice failed to alter their rate of nose poking in response to a change in concentration. The lack of concentration-dependent responding in XX/*Sry* + mice may reflect differences in their ability to flexibly update their behavior or differences in EtOH reward. Although it is not clear at this time why XX/*Sry* + mice differed from the other genotypes on this task, this particular result highlights the fact that sex chromosome effects can be dependent on gonad type.

In the second operant experiment, FCG mice did not reduce responding when quinine was added to the EtOH solution and no differences were seen between any of the genotypes. Although this result was unexpected considering that we have previously reported that concentrations of quinine above 250 μM reduce responding for EtOH in male C57BL/6 J mice (24), it can be explained by the quinine insensitivity observed in the *Sry* + mice. The *Sry–* mice performed similarly to what we have previously observed in C57BL/6 J mice (24) and the lack of a difference between XX and XY animals of this gonad type suggests that chromosomal sex is not a major determinant of aversion-resistant responding under these experimental conditions. While this result differs from what was observed in the DID paradigm, where the XY complement increased aversion-resistance in *Sry–* mice, it is important to consider that female mice demonstrate quinine-resistance at higher quinine concentrations in the operant task than in the limited access DID model. When testing C57BL/6 J females, we have demonstrated that they decrease consumption at 250 and 500 μM quinine concentrations when drinking in the home cage (27) but continue responding for EtOH adulterated with these same concentrations in the operant chamber (24). Thus, the lack of an effect of sex chromosomes in *Sry–* mice in this experiment is likely due to a ceiling effect.

Because the results with quinine were inconclusive in *Sry* + mice, and to determine whether the *Sry* + mice are resistant to other punishments, we tested a subset of mice with a 0.35 mA footshock. We have previously found that this amplitude of footshock reduces responding for EtOH in male but not female C57BL/6 J mice (56) as well in both sexes in the selectively bred crossed High Alcohol Preferring line of mice (57). As all FCG mice reduced their responding on the footshock session, we conclude that any insensitivity to punishment in this line may be specific to quinine, although this portion of the study should be considered exploratory considering that only a subset of mice were tested. Future studies could use footshock to more fully explore whether aversion-resistance is regulated by sex chromosomes in the operant self-administration task.

In addition to responding for EtOH in the operant task, we also assessed head entries into the reward receptacle, which could be considered a measure of seeking behavior. While the number of head entries was similar between the two operant experiments, the findings were unexpectedly different. In the concentration experiment, *Sry* + mice made more head entries than *Sry–* mice (*p* = 0.0142). This effect of gonadal sex was not observed in the quinine experiment and instead XX mice made more head entries than XY mice (*p* = 0.049). Similar to the nose poke data, head entries did not decrease with quinine concentration. Persistent head entries, along with our observation that the drinking cups were empty at the end of the experiment, demonstrate that mice continued to consume quinine-adulterated EtOH at even the highest concentrations. Head entry data also followed nose poke responses in the subset of mice administered footshock, decreasing on the footshock session when compared to baseline. Thus, head entry data further support the conclusion that FCG mice are insensitive to quinine, but not footshock, punishment.

For the operant experiments, it is important to note that many mice, particularly those with the XY chromosome complement, failed to reach the training criterion and were dropped from the study. Of the 18 mice that did not reach criterion in the quinine-resistance experiment, 50% were XY/*Sry–* and 28% were XY/*Sry*+. This observation may point to differences in the genetic background of the FCG mice vs. lines that readily learn the operant response task (i.e., C57BL/6 J and crossed High Alcohol Preferring mice; see 23, 39, 40) or changes in the Y chromosome resulting from deletion of the *Sry* gene in XY mice specifically. Because of the high dropout rate, it is possible that this experiment is underpowered and indeed, there were some interesting trends in the data that did not reach significance (e.g., higher responding in XX/*Sry* + vs. XY/*Sry* + mice). However, between the difficulty in training XY mice and the demonstrated quinine insensitivity of the *Sry* + animals, it would be difficult to draw additional meaningful conclusions from this experiment even if more subjects were tested.

## 5. Conclusion

The current results provide evidence that sex chromosome complement influences EtOH consumption and preference in a limited access DID model, with XY chromosomes influencing intake in mice with testes (*Sry*+) and preference in mice of both gonadal types. We further demonstrate that XY chromosomes promote aversion-resistance in mice with ovaries (*Sry–*). Finally, sex chromosomes influence responding for EtOH in an operant task in a concentration-dependent manner. Importantly, these effects are not influenced by the estrous cycle or sensitivity to the sedative effects EtOH, as no differences were observed in latency to lose the righting reflex or the duration of the LORR between genotypes. Similarly, we found no differences in EtOH concentration in the blood between any of the genotypes. These findings add to a growing body of literature suggesting that chromosomal sex may be an important contributor to alcohol drinking behaviors.

A limitation of this study is that all mice had freely cycling hormones, and, therefore, we cannot be certain that the effects seen here are solely chromosomal. Indeed, the fact that some sex chromosome effects occur only in *Sry–* or *Sry* + mice suggests that these effects are dependent on gonad type and the behaviors under study likely involve interactions between sex hormones and sex chromosomes. Gonadal influences are likely to be primarily organizational (i.e., occurring during development; (58)), as we did not see an influence of the estrous cycle on aversion-resistant EtOH drinking. Similar studies in gonadectomized mice would need to be conducted to concretely rule out any ongoing influence of sex hormones on these behaviors, though we note that one study in FCG mice found no influence of gonadectomy on EtOH consumption or habitual responding (19). We were also unable to assess the contribution of *Sry* to home cage drinking behaviors since we tested the *Sry–* and *Sry +* groups separately and monitored estrous cycle *via* vaginal lavage in *Sry–* mice. As estrous cycle monitoring can only occur in females, we followed recommendations to make comparisons within cohorts based on gonadal sex (9, 54).

It will be important for future studies to investigate exactly how sex chromosomes influence the development of alcohol drinking behaviors. There may be functional differences in the XX vs. XY chromosome complements resulting from the addition of Y genes or a double dose of some X genes (18). The current results suggest that genes on the Y chromosome may confer some vulnerability to binge-like and aversion-resistant drinking or that genes on the X chromosome may protect against these behaviors. As an example, *NlGN4X*, a gene on the X chromosome, has been associated with alcohol dependence in men only (59) and may be a candidate gene for increasing the risk of developing AUD. Another recent study has found that following prenatal EtOH exposure in females, the X chromosome inactivation factor *Xist* was downregulated and *lncRNA Tsix,* which inhibits *Xist,* was upregulated (along with other X chromosomes genes) (60). These data suggest that EtOH exposure may result in the loss of X chromosome inactivation and may be linked with pathological drinking behaviors. Future mechanistic studies into how sex chromosomes regulate alcohol drinking behaviors may yield other novel targets for therapeutic intervention.

## Data availability statement

The raw data supporting the conclusions of this article will be made available by the authors, without undue reservation.

## Ethics statement

The animal study was reviewed and approved by Institutional Animal Care and Use Committee at Miami University.

## Author contributions

ES, AR, JD, MC, BH, and SP designed the experiments. ES, BM, KR, JD, MC, BH, and SP conducted the experiments. ES, AR, BM, KR, JD, MC, BH, and SP analyzed the data. ES and AR interpreted the findings. ES, AR, KR, and CT drafted the manuscript and figures. All authors contributed to the article and approved the submitted version. The home cage drinking and LORR studies included in this manuscript were previously in Dr. Sneddon’s dissertation (Sneddon, 2022).

## Funding

This work was supported by NIH grants R15 AA027915 (AR) and F99 NS118727 (ES), the Office of Research for Undergraduates (BM, JD, MC, BH, and SP), the Office of Research and Innovation, and the Broadening Undergraduate Research Participation Program in the Department of Psychology at Miami University.

## Conflict of interest

The authors declare that the research was conducted in the absence of any commercial or financial relationships that could be construed as a potential conflict of interest.

## Publisher’s note

All claims expressed in this article are solely those of the authors and do not necessarily represent those of their affiliated organizations, or those of the publisher, the editors and the reviewers. Any product that may be evaluated in this article, or claim that may be made by its manufacturer, is not guaranteed or endorsed by the publisher.
